# Dietary quality is associated with lower postpartum weight retention among women with prior gestational diabetes in a sub-high altitude region of China

**DOI:** 10.3389/fnut.2026.1870999

**Published:** 2026-09-03

**Authors:** Jiahui He, Na Wang, Jianqin Yang, Qian Zhao, Yan Tian, Lei Shu, Li Li, Shuhua Qian

**Affiliations:** 1 Department of Neonatology, Obstetrics & Gynecology Hospital of Fudan University, Shanghai Key Lab of Reproduction and Development, Shanghai Key Lab of Female Reproductive Endocrine Related Diseases, Shanghai, China; 2 Department of Obstetrics, Obstetrics & Gynecology Hospital of Fudan University, Shanghai Key Lab of Reproduction and Development, Shanghai Key Lab of Female Reproductive Endocrine Related Diseases, Shanghai, China; 3 Department of Nursing, Yongping County People’s Hospital, Dali, China; 4 Department of Emergency, Yongping County People’s Hospital, Dali, China; 5 Department of Nutrition, Obstetrics & Gynecology Hospital of Fudan University, Shanghai Key Lab of Reproduction and Development, Shanghai Key Lab of Female Reproductive Endocrine Related Diseases, Shanghai, China

**Keywords:** dietary quality, gestational diabetes mellitus, maternal health, nutritional epidemiology, physical activity, postpartum weight retention, sub-high altitude

## Abstract

**Background:**

Women with a history of gestational diabetes mellitus (GDM) are at high risk of long-term metabolic disorders, and postpartum weight retention (PPWR) may further accelerate this progression. However, evidence regarding the independent roles of dietary quality and physical activity in sub-high altitude regions remains limited. This study aimed to evaluate the associations between lifestyle behaviors and PPWR among women with prior GDM living in southwestern China.

**Methods:**

This cross-sectional study included 141 women with a history of GDM who were 1–3 years postpartum and residing in Dali, a sub-high altitude region of China. Dietary quality was assessed using a localized semi-quantitative food frequency questionnaire, and physical activity was evaluated using the International Physical Activity Questionnaire–Short Form. Multivariable linear regression models were used to identify independent correlates of continuous PPWR after adjustment for demographic, obstetric, and lifestyle covariates. Sensitivity analysis was performed using binary logistic regression with clinically significant PPWR (≥5 kg) as the outcome.

**Results:**

Higher dietary quality was independently associated with lower PPWR in the fully adjusted model (*B* = −0.099, 95% CI: −0.175 to −0.023, *p* = 0.011). Each one-point increase in dietary quality score was associated with an approximately 0.1 kg lower PPWR; equivalently, a 10-point increase in dietary quality score was associated with an approximately 1.0 kg reduction in PPWR. This association remained stable in sensitivity analysis (OR = 0.937, 95% CI: 0.885–0.991, *p* = 0.022). Although physical activity showed an inverse association with PPWR in univariate analysis, it was no longer statistically significant after multivariable adjustment. Lower pre-pregnancy body mass index and higher neonatal birth weight were also independently associated with greater PPWR.

**Conclusion:**

Among women with prior GDM living in a sub-high altitude region, better dietary quality was independently associated with lower long-term PPWR, whereas self-reported physical activity was not. These findings support region-specific dietary counseling as a potentially important strategy for postpartum metabolic risk reduction in high-risk women.

## 1 Introduction

Gestational diabetes mellitus (GDM) is a major global public health concern, affecting approximately 14% of pregnancies worldwide (1). Although maternal glycemic control typically normalizes shortly after parturition, the underlying metabolic perturbations frequently persist. Women with a history of GDM have an approximately tenfold higher risk of developing type 2 diabetes mellitus (T2DM) compared to their normoglycemic counterparts (2). Within this trajectory, the postpartum period constitutes a critical “window of opportunity” for metabolic reset, yet it is simultaneously a period of profound vulnerability. Long-term postpartum weight retention (PPWR), which is defined as the sustained failure to return to pre-pregnancy weight beyond the first year and extending into subsequent years following delivery, is a key modifiable factor that contributes to this adverse metabolic trajectory (3). Physiologically, excessive PPWR, characterized by the accumulation of visceral adipose tissue, promotes a chronic pro-inflammatory state and impairs adipokine signaling (4). These metabolic disturbances exacerbate insulin resistance and drive a progressive decline in β-cell compensatory function, thereby accelerating the transition from GDM to type 2 diabetes even years after delivery (5). Consequently, identifying and mitigating the determinants of PPWR in this high-risk population is paramount to disrupting the pathogenic cascade from GDM to chronic metabolic disease (6).

Lifestyle modification remains central to postpartum weight management and cardiometabolic risk reduction. Contemporary clinical guidelines advocate for the synergistic implementation of dietary optimization and physical activity; however, the relative independent contributions of these behaviors to PPWR remain unclear (7). While physical activity is universally recommended to increase energy expenditure, postpartum women face profound structural and physiological barriers to sustained exercise, including fragmented sleep, fatigue, and overwhelming childcare demands (8). Specifically, evidence from GDM populations suggests that device-measured physical activity levels often fall short of necessary metabolic thresholds during the first 2 years postpartum, despite self-reported intentions (9). Conversely, recent epidemiological evidence suggests that dietary quality, which evaluates the cumulative and synergistic effects of overall food intake patterns rather than isolated macronutrients, may exert a more sustained influence on long-term maternal weight regulation, particularly among women with a history of GDM (10). High-quality dietary patterns, characterized by high intakes of fresh vegetables, soy products, and fungi/algae, have been inversely associated with postpartum weight retention in regional Chinese cohorts (11). Nevertheless, the preponderance of existing literature focuses on general postpartum populations, and evidence specifically disentangling the independent associations of dietary quality versus physical activity among the vulnerable post-GDM subgroup remains scarce. Understanding these specific behavioral drivers is essential for tailoring precision public health interventions that transcend generic lifestyle advice.

Beyond these behavioral drivers, the geographical and environmental context significantly modulates both lifestyle patterns and metabolic regulation. Despite the expanding evidence base concerning PPWR, a notable environmental gap persists; the overwhelming majority of studies have been conducted in lowland regions or Western populations, leaving unique geographical settings, such as sub-high altitude areas (typically 1,500–3,000 meters above sea level), markedly underrepresented (10, 11). This gap is critically important because environmental constraints and regional cultural paradigms profoundly shape metabolic responses and dietary choices. In the sub-high altitude regions of southwestern China, for instance, dietary structures frequently demonstrate a heavy reliance on refined carbohydrates and a constrained consumption of diverse fresh vegetables, paralleling patterns observed in higher-altitude Tibetan populations (12). These dietary shifts are driven by shared agricultural and climatic limitations. Furthermore, chronic exposure to sub-high altitude hypoxic environments can modulate energy homeostasis. Evidence from high-altitude physiology suggests a metabolic shift favoring carbohydrate oxidation over lipid oxidation (13), a trend that may manifest even at moderate elevations and fundamentally alter the efficacy of standard lipid-mobilization-dependent weight loss strategies in sub-high altitude cohorts. Additionally, reduced maximal oxygen uptake characteristic of these elevations exacerbates physiological barriers to sustained high-intensity physical activity (14). This reduction in aerobic capacity, coupled with the aforementioned metabolic shifts, may diminish the overall contribution of exercise to energy balance, thereby making dietary quality a potentially more critical and actionable determinant of weight retention in sub-high altitude cohorts than in their lowland counterparts. To date, however, no study has specifically examined how these unique environmental and culturally ingrained lifestyle patterns impact PPWR among the vulnerable post-GDM population.

Therefore, this study aimed to evaluate the independent and combined associations of dietary quality, physical activity, and sedentary behavior with PPWR among women with a history of GDM living in a sub-high altitude region of China. Using multivariable linear regression models with adjustment for key obstetric and demographic confounders, including pre-pregnancy BMI and gestational weight gain (GWG), we sought to clarify the relative contributions of diet and movement to postpartum weight trajectories.

## 2 Materials and methods

### 2.1 Study design and participants

This cross-sectional study was conducted at Yongping County People’s Hospital (Dali Prefecture, Yunnan Province, China), a facility located within a characteristic sub-high altitude region. Participants were recruited between December 15, 2025, and January 15, 2026, via structured telephone follow-up.

Eligible participants were women with a documented history of gestational diabetes mellitus (GDM) who delivered at the study site between January 1, 2023, and December 31, 2024. GDM was diagnosed during pregnancy based on the International Association of Diabetes and Pregnancy Study Groups (IADPSG) criteria (15).

**FIGURE 1:**
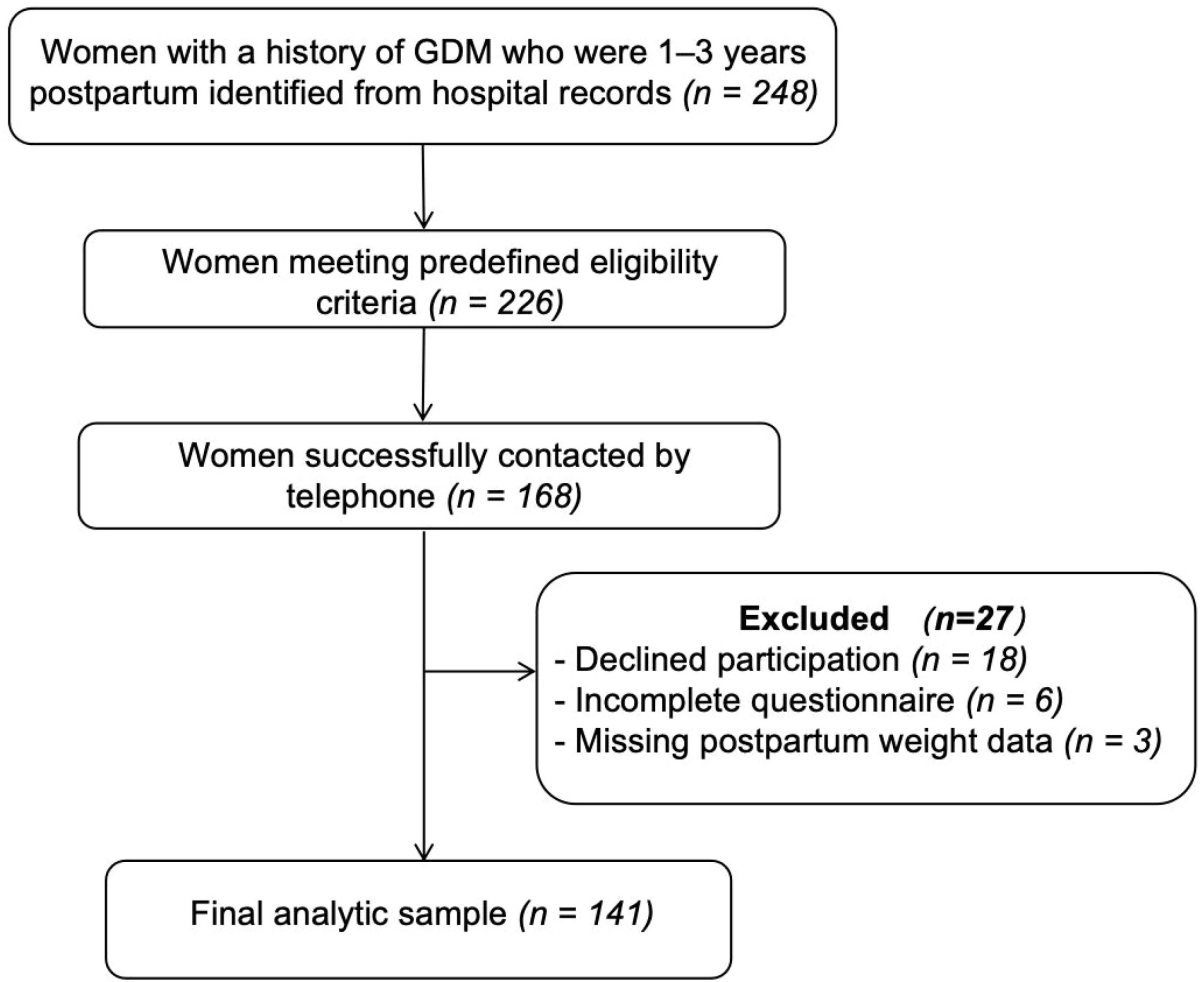
Flow diagram of participant recruitment and inclusion process.

Inclusion criteria were: (1) confirmed GDM diagnosis; (2) singleton pregnancy; (3) full-term delivery (≥37 gestational weeks); (4) availability of comprehensive baseline obstetric records. Exclusion criteria comprised: (1) pre-existing type 1 or type 2 diabetes; (2) severe maternal comorbidities (e.g., end-stage renal disease, severe cardiovascular pathology); (3) multiple gestation; (4) fetal congenital anomalies; (5) severe placental or umbilical cord complications; (6) fetal growth restriction, stillbirth, or neonatal death; and (7) any subsequent pregnancy prior to the follow-up assessment. Eligible women were contacted by telephone and invited to participate. Women who agreed to participate provided verbal informed consent before completing the questionnaire and follow-up assessment. A final analytic sample of 141 women was included in the present study. The participant selection process is shown in Figure 1. The study protocol was approved by the Ethics Committee of Yongping County People’s Hospital (approval number: ypxrmyy1120250002), and all procedures adhered to the Declaration of Helsinki.

### 2.2 Data collection and measurements

Data were collected through structured telephone interviews conducted by trained investigators using standardized instruments. Baseline obstetric and neonatal data were extracted from hospital medical records to ensure accuracy and minimize recall bias.

#### 2.2.1 Dietary assessment

Dietary quality was evaluated using a semi-quantitative Food Frequency Questionnaire (FFQ) adapted from the Chinese Expert Consensus and Group Standard for Weight Management in Overweight or Obese Populations (16). The questionnaire was based on a previously established national dietary assessment framework, and minor modifications were made to reflect locally consumed staple foods and dietary patterns in the study region. Specifically, four items (Q4, Q12, Q17, and Q21) were localized to enhance regional specificity and capture local dietary characteristics, while maintaining the original food classification structure, frequency response options, and scoring algorithm. The dietary quality domains and scoring principles were retained from the original framework to ensure methodological consistency. The total Dietary Quality Score (DQS) ranged from 0 to 100, with higher scores indicating greater dietary quality, nutritional adequacy, and dietary diversity.

#### 2.2.2 Physical activity and sedentary behavior

Physical activity was quantified using the Chinese version of the International Physical Activity Questionnaire–Short Form (IPAQ-SF) (17), which has been previously validated for use in Chinese populations. Participants reported the frequency and duration of vigorous-intensity activity, moderate-intensity activity, and walking over the preceding 7 days. Total physical activity was calculated in metabolic equivalent of task-hours per week (MET-h/week) according to the standard IPAQ scoring protocol (18). Sedentary behavior was quantified as self-reported daily sitting time (h/day).

#### 2.2.3 Anthropometric and obstetric covariates

Sociodemographic variables included age, ethnicity (Han or minority), educational attainment, and occupation (agricultural vs. non-agricultural). Obstetric characteristics included parity, gestational age at delivery, mode of delivery (vaginal vs. cesarean section), and neonatal birth weight. Pre-pregnancy Body Mass Index (BMI) was calculated using self-reported pre-pregnancy weight divided by the square of height (kg/m^2^). Gestational weight gain (GWG) was determined as the difference between maternal weight at delivery and pre-pregnancy weight, following the standard calculation procedures described by the Institute of Medicine (IOM) (19). Gestational weight gain was included as a continuous variable in the regression models to evaluate its potential linear association with postpartum weight retention. Given the limited sample size, GWG was not categorized according to Institute of Medicine (IOM) recommendations to avoid loss of statistical power and unstable estimates. Breastfeeding data referred to the duration of exclusive breastfeeding (months).

### 2.3 Definition of variables

The primary outcome was postpartum weight retention (PPWR), defined as the difference between current weight at follow-up and pre-pregnancy weight (kg). PPWR was analyzed as a continuous variable in the primary models. For sensitivity analysis, PPWR was dichotomized into “significant retention” (≥5 kg) and “minimal retention” (<5 kg). Although the 5 kg threshold is traditionally validated for the 12-month postpartum milestone (20), its application in the current 1–3 year postpartum cohort serves as a conservative indicator of persistent adiposity that escaped early postpartum resolution. Primary exposures included the DQS, total physical activity (MET-h/week), and daily sitting time. Covariates included maternal age, ethnicity, education, occupation, pre-pregnancy BMI, GWG, parity, gestational age, delivery mode, neonatal weight, breastfeeding duration, and follow-up interval.

### 2.4 Statistical analysis

Analyses were performed using SPSS version 27.0 (IBM Corp., Armonk, NY, USA). A two-tailed *P* < 0.05 defined statistical significance. Continuous variables were expressed as mean ± standard deviation (SD) for normally distributed data or median with interquartile range (IQR) for non-normally distributed data. Categorical variables were presented as frequencies and percentages (*n*, %).

Group comparisons between women with PPWR ≥ 5 kg and <5 kg utilized independent-sample *t*-tests, Mann–Whitney U tests, or chi-square tests, as appropriate.

Univariate linear regression explored factors associated with continuous PPWR. Subsequently, multivariable linear regression analysis was performed to identify independent factors associated with PPWR. Variables were selected based on clinical relevance and previous literature. Follow-up duration was included as a covariate in the multivariable regression models to account for potential differences in postpartum timing. Multicollinearity was assessed via variance inflation factors (VIF < 5). Model assumptions were rigorously evaluated through residual normality (P–P plots), homoscedasticity (standardized residual scatter plots), independence of errors (Durbin–Watson statistic), and influential observations (Cook’s distance). To assess the robustness of the findings, a sensitivity analysis using binary logistic regression was conducted with clinically significant PPWR (≥5 kg) as the dependent variable. Variables included in this model were selected *a priori* based on clinical relevance and findings from the primary multivariable analysis. The study was reported in accordance with the STROBE statement.

## 3 Results

### 3.1 Study population characteristics

The analysis included 141 postpartum women with a documented history of gestational diabetes mellitus (GDM). Participants were categorized according to weight retention status: 21 (14.9%) had significant retention (PPWR ≥ 5 kg), whereas 120 (85.1%) showed minimal retention (PPWR < 5 kg).

As shown in Table 1, significant between-group differences were observed in occupation (*P* = 0.038), educational level (*P* < 0.001), pre-pregnancy body mass index (BMI; *P* = 0.016), gestational weight gain (GWG; *P* = 0.033), and follow-up interval (*P* = 0.018). Dietary quality scores were significantly lower in the significant retention group than in the minimal retention group (median: 52.0 vs. 56.0, *P* = 0.005).

**TABLE 1:** Characteristics of women with postpartum weight retention <5 kg and ≥5 kg (*N* = 141).

Variable	PPWR < 5 kg (*n* = 120)	PPWR ≥ 5 kg (*n* = 21)	*P*-value
Maternal age (years)	30.4 ± 4.5	30.4 ± 4.5	0.956
Ethnicity			0.304
Han	77 (64.2)	11 (52.4)	
Minority	43 (35.8)	10 (47.6)	
Occupation			0.038
Farmer	82 (68.3)	19 (90.5)	
Non-agricultural occupation	38 (31.7)	2 (9.5)	
Education level			<0.001
Junior high school or below	38 (31.7)	3 (14.3)	
Technical secondary school or senior high school	17 (14.2)	17 (81.0)	
College or university	65 (54.2)	1 (4.8)	
Gestational age at delivery (weeks)	39.1 (38.4–39.7)	39.1 (38.7–39.6)	0.754
Parity			0.896
Primipara	59 (49.2)	10 (47.6)	
Multipara	61 (50.8)	11 (52.4)	
Delivery mode			0.612
Vaginal delivery	47 (39.2)	7 (33.3)	
Cesarean section	73 (60.8)	14 (66.7)	
Pre-pregnancy BMI (kg/m^2^)	25.3 ± 4.1	23.0 ± 3.8	0.016
Gestational weight gain (kg)	10.9 ± 5.1	13.6 ± 5.9	0.033
Neonatal birth weight (g)	3239.9 ± 448.9	3307.1 ± 296.5	0.509
Follow-up duration (months)	23.0 (15.0–30.0)	30.0 (22.0–32.0)	0.018
Exclusive breastfeeding duration (months)	3.0 (0–6.0)	6.0 (0–6.0)	0.507
Dietary quality score	56.0 (52.0–63.0)	52.0 (47.0–55.0)	0.005
Physical activity (MET-h/week)	1386.0 (693.0–2772.0)	693.0 (396.0–2079.0)	0.167
Sitting time (h/day)	3.0 (2.0–5.5)	5.0 (2.0–7.0)	0.077

Data are presented as mean ± standard deviation (SD) for normally distributed continuous variables, median (interquartile range, IQR) for non-normally distributed continuous variables, and number (percentage, %) for categorical variables. *P*-values were calculated using the independent-samples *t*-test, Mann–Whitney U test, or chi-square test, as appropriate. GDM, gestational diabetes mellitus; BMI, body mass index; GWG, gestational weight gain; PPWR, postpartum weight retention; PA, physical activity; MET, metabolic equivalent.

No significant differences were found for maternal age, ethnicity, gestational age at delivery, parity, delivery mode, neonatal birth weight, exclusive breastfeeding duration, physical activity, or sitting time (all *P* > 0.05).

### 3.2 Univariate correlates of PPWR

Univariate linear regression showed that educational level, GWG, and sitting time were positively associated with continuous PPWR (all *P* < 0.05). In contrast, pre-pregnancy BMI (*B* = −0.533, *P* < 0.001), dietary quality score (*B* = −0.177, *P* < 0.001), and physical activity (*B* = −0.001, *P* = 0.003) were inversely associated with PPWR (Table 2).

**TABLE 2:** Univariate linear regression analysis of factors associated with postpartum weight retention.

Variable	B	SE	β	t	*P*
Maternal age (years)	−0.019	0.094	−0.017	−0.203	0.840
Ethnicity (minority vs. Han)	1.713	0.887	0.162	1.931	0.055
Occupation (non-agricultural occupation vs. farmer)	−0.807	0.963	−0.071	−0.838	0.403
Education level					
Junior high school or below	–	–	–	–	(Ref)
Technical secondary school or senior high school	4.885	1.100	0.407	4.441	**<0.001**
College or university	0.048	0.943	0.005	0.051	0.960
Gestational age at delivery (weeks)	0.468	0.467	0.085	1.004	0.317
Parity (multipara vs. primipara)	−1.265	0.864	−0.123	−1.464	0.145
Delivery mode (cesarean section vs. vaginal delivery)	1.205	0.889	0.114	1.354	0.178
Pre-pregnancy BMI (kg/m^2^)	−0.533	0.096	−0.427	−5.566	**<0.001**
Gestational weight gain (kg)	0.265	0.079	0.273	3.343	**0.001**
Neonatal birth weight (g)	0.001	0.001	0.118	1.405	0.162
Follow-up duration (months)	0.051	0.054	0.081	0.961	0.338
Exclusive breastfeeding duration (months)	0.184	0.154	0.101	1.197	0.233
Dietary quality score	−0.177	0.043	−0.329	−4.110	**<0.001**
Physical activity (MET-h/week)	−0.001	0.000	−0.251	−3.062	**0.003**
Sitting time (h/day)	0.484	0.171	0.233	2.828	**0.005**

Univariate linear regression analyses were performed to examine the associations between individual independent variables and postpartum weight retention (kg). Unstandardized coefficients (B), standard errors (SE), standardized coefficients (β), *t*-values, and *P*-values are presented. Statistical significance was defined as *P* < 0.05. Significant values are presented in bold.

**FIGURE 2:**
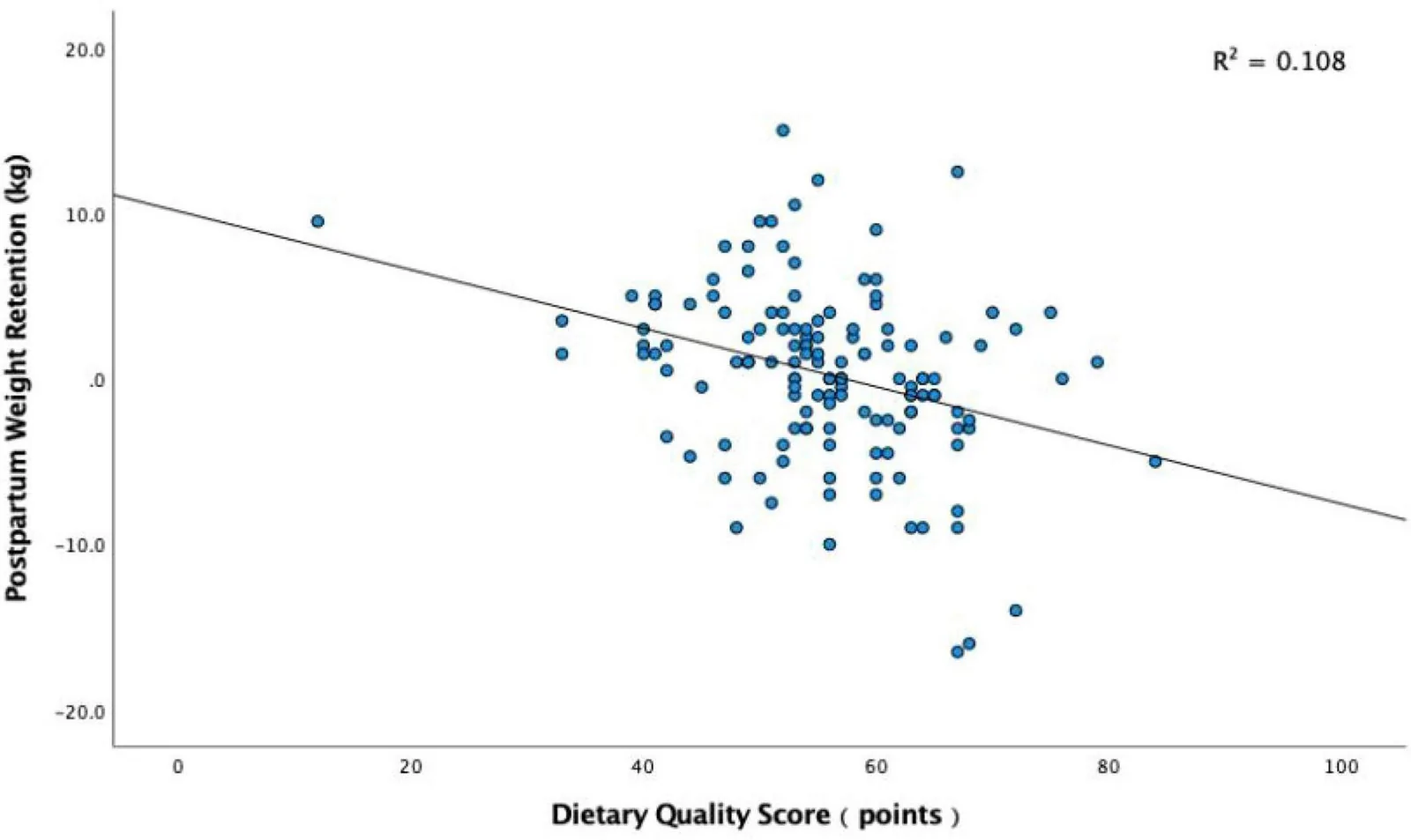
Association between regional dietary quality score and postpartum weight retention (kg). The scatter plot illustrates an inverse relationship between dietary quality score and postpartum weight retention. The solid line represents the fitted linear regression line (R^2^ = 0.108, *p* < 0.05), and the shaded area indicates the 95% confidence interval.

Scatter plot analysis further supported this association, demonstrating a negative linear relationship between dietary quality and PPWR (R^2^ = 0.108; Figure 2).

### 3.3 Multivariate hierarchical regression analysis

To identify independent factors associated with PPWR, a multivariable linear regression model was constructed including demographic, obstetric, and lifestyle-related variables. The final model explained 47.4% of the variance in PPWR (R^2^ = 0.474; adjusted R^2^ = 0.411, *F* = 7.519, *P* < 0.001).

As shown in Table 3, each one-point increase in DQS was associated with a 0.099 kg reduction in PPWR (*B* = –0.099, 95% CI: –0.175 to –0.023, *P* = 0.011). Given the distribution of DQS in this cohort (mean ± SD: 55.63 ± 9.59), a one-standard-deviation increase in DQS (9.59 points) corresponded to an estimated reduction of approximately 0.95 kg in PPWR, which was comparable to the reduction associated with a 10-point increase in DQS (∼0.99 kg).

**TABLE 3:** Multivariable linear regression analysis of independent predictors for postpartum weight retention.

Variables	B	SE	β	t	*P*	95% CI	VIF
Age (years)	0.142	0.086	0.128	1.641	0.103	−0.029 to 0.313	1.449
Pre-pregnancy BMI (kg/m^2^)	−0.476	0.100	−0.382	−4.774	**<0.001**	−0.674 to −0.279	1.520
Gestational weight gain (kg)	0.014	0.077	0.015	0.185	0.853	−0.139 to 0.167	1.505
Gestational age at delivery (weeks)	0.142	0.447	0.026	0.317	0.752	−0.743 to 1.027	1.559
Follow-up duration (months)	−0.057	0.045	−0.091	−1.266	0.208	−0.147 to 0.032	1.221
Neonatal birth weight (g)	0.002	0.001	0.175	2.028	**0.045**	0.000–0.004	1.779
Exclusive breastfeeding duration (months)	0.222	0.131	0.122	1.695	0.093	−0.037 to 0.481	1.228
Dietary quality score	−0.099	0.038	−0.184	−2.578	**0.011**	−0.175 to −0.023	1.210
Physical activity (MET-h/week)	0.000	0.000	−0.117	−1.526	0.129	−0.001 to 0.000	1.391
Sitting time (h/day)	−0.044	0.159	−0.021	−0.279	0.781	−0.358 to 0.270	1.387
Education level							
Primary or below (ref)	–	–	–	–	–	–	
Middle school	3.709	0.989	0.309	3.749	**<0.001**	1.751–5.666	1.618
High school or above	−1.069	0.883	−0.104	−1.211	0.228	−2.817 to 0.678	1.754
Ethnicity							
Han (ref)	–	–	–	–	–	–	
Minority	1.153	0.736	0.109	1.566	0.120	−0.304 to 2.610	1.148
Parity							
Primipara (ref)	–	–	–	–	–	–	
Multipara	−1.666	0.850	−0.162	−1.959	0.052	−3.348 to 0.017	1.631
Delivery mode							
Vaginal delivery (ref)	–	–	–	–	–	–	
Cesarean section	1.119	0.773	0.106	1.447	0.150	−0.411 to 2.649	1.276

The multivariable model simultaneously adjusted for demographic, obstetric, follow-up, and lifestyle variables listed in the table. The unstandardized coefficient (B) represents the estimated change in postpartum weight retention (kg) associated with a one-unit increase in each independent variable. Statistical significance was defined as *P* < 0.05. Model statistics: R^2^ = 0.474; adjusted R^2^ = 0.411; *F* = 7.519; *P* < 0.001. Bold values indicate statistical significance (*P* < 0.05). BMI, body mass index; SE, standard error; CI, confidence interval.

Pre-pregnancy BMI also remained inversely associated with PPWR (*B* = −0.476, *P* < 0.001), whereas neonatal birth weight was positively associated with PPWR (*B* = 0.002, *P* = 0.045). Education level was also associated with PPWR, with participants with middle school education showing higher PPWR compared with those with primary education or below (*B* = 3.709, *P* < 0.001).

After multivariable adjustment, physical activity (*P* = 0.129) and sitting time (*P* = 0.781) were not independently associated with PPWR.

### 3.4 Model diagnostics and sensitivity analysis

Model diagnostics indicated that all assumptions for linear regression were adequately met. No evidence of multicollinearity was detected (all VIF < 2), and the Durbin–Watson statistic was 2.207, suggesting no residual autocorrelation.

Residual P–P plots and standardized residual scatter plots supported normality and homoscedasticity assumptions. In addition, the maximum Cook’s distance was 0.081, indicating no influential outliers.

In the sensitivity analysis, binary logistic regression was performed with clinically significant PPWR (≥5 kg) as the dependent variable. Based on clinical relevance and the primary multivariable analysis, the model included dietary quality score, total physical activity, sitting time, pre-pregnancy BMI, and gestational weight gain. Dietary quality remained independently associated with lower odds of significant PPWR (OR = 0.937, 95% CI: 0.885–0.991, *P* = 0.022). These results were consistent with those of the primary linear regression analysis.

## 4 Discussion

This study investigated the independent associations between lifestyle behaviors and postpartum weight retention (PPWR) among women with a history of gestational diabetes mellitus (GDM) residing in a sub-high altitude region of China. Our primary finding demonstrated that, after adjustment for a comprehensive set of demographic and obstetric confounders, dietary quality remained an independent factor associated with lower PPWR. Notably, while physical activity (PA) exhibited a significant inverse association with PPWR in univariate models, this association was attenuated after multivariate adjustment. These findings suggest that, within this specific demographic and environmental context, dietary quality may play a more prominent role in postpartum weight trajectories than physical activity.

### 4.1 Mechanistic insights: the dominance of dietary quality over physical activity

Several mechanisms may partially explain the observed associations. High-quality dietary patterns, particularly those rich in dietary fiber and low-glycemic index (GI) foods, may contribute to improved glucose homeostasis and insulin sensitivity, which are often impaired in the post-GDM state (21, 22). In addition, fiber-rich diets may enhance satiety and influence the gut microbiota, thereby potentially reducing systemic inflammation associated with adipose tissue accumulation (23, 24).

Previous studies have demonstrated that physical activity is an important determinant of postpartum weight trajectories. Higher levels of postpartum physical activity have been associated with greater weight loss or reduced weight retention in several populations, including overweight and obese women and women participating in lifestyle intervention programs (25, 26). However, the contribution of physical activity may vary depending on population characteristics, measurement methods, and postpartum living environments. Conversely, the attenuation of PA in the multivariate models may reflect the “fragmented movement barrier” commonly observed during the postpartum period (27). Unlike structured exercise, postpartum PA is often characterized by intermittent childcare tasks and household activities. Emerging evidence suggests that such fragmented movement may not consistently reach the metabolic equivalent (MET) thresholds or sustained intensity required to meaningfully improve oxidative capacity or promote fat metabolism (28, 29). Furthermore, in sub-high altitude environments such as Dali, hypoxia-related physiological stress may increase perceived exertion during physical activity, potentially limiting both duration and intensity compared with lowland settings (30, 31). Taken together, these factors may reduce the relative contribution of PA to long-term energy balance, whereas dietary modification may represent a more feasible behavioral target during the 1–3-year postpartum period.

### 4.2 The compensatory weight retention paradox and obstetric cascade

We observed a significant inverse association between pre-pregnancy BMI and PPWR, which is consistent with the concept of a “compensatory weight retention paradox” (32). Women with lower pre-pregnancy BMI may undergo greater physiological adaptations toward energy storage during pregnancy, possibly as a mechanism to support lactation, which could contribute to increased postpartum weight retention (33). In addition, clinical attention is often more focused on overweight or obese women during pregnancy, and those with a normal baseline BMI may receive comparatively less targeted guidance on postpartum weight management (34). This difference in clinical attention may partially influence postpartum behavioral patterns.

The positive association between neonatal birth weight and PPWR observed in this study may also reflect a broader metabolic continuum. Higher birth weight is often linked to excessive gestational weight gain (GWG) and maternal hyperglycemia, both of which may contribute to sustained postpartum adiposity through hormonal and inflammatory pathways (35–37). These findings are in line with previous studies suggesting that maternal weight regulation should be considered across the entire continuum from preconception to the postpartum period (38, 39).

### 4.3 Environmental influences: sub-high altitude and regional dietary patterns

An important strength of this study is its focus on a sub-high altitude population, which has been relatively underrepresented in the literature. In southwestern China, dietary patterns are often characterized by a high reliance on carbohydrate-rich staple foods such as rice and tubers, influenced by regional agricultural and cultural factors (40, 41). In this context, a higher dietary quality score may reflect greater dietary diversity and improved nutrient balance.

Additionally, chronic exposure to moderate hypoxia may influence substrate utilization, with some evidence suggesting a relative shift toward carbohydrate metabolism (13, 42). Under such conditions, diets high in refined carbohydrates may have more pronounced metabolic effects among women with a history of GDM. Our findings differ from those reported in lowland urban populations, where sedentary behavior and Westernized dietary patterns are more commonly identified as primary contributors to weight retention (43). This contrast highlights the importance of considering environmental and cultural context when interpreting lifestyle–health relationships.

### 4.4 Public health and clinical implications: precision nutrition

These findings may have implications for public health strategies targeting women with a history of GDM. The commonly recommended approach of “eat less and move more” may not fully capture the complexities faced by women in sub-high altitude or resource-limited settings (44, 45). Instead, regionally tailored nutritional strategies that focus on improving dietary quality may be more practical and acceptable.

Such approaches could include increasing the intake of high-quality protein sources, vegetables, and micronutrient-rich foods, while addressing the reliance on refined carbohydrate staples. Incorporating culturally appropriate dietary counseling into routine postpartum care may help support sustainable behavior change and reduce long-term metabolic risk in this population (7, 46).

### 4.5 Strengths and limitations

This study has several strengths, including the use of multivariate models to account for key demographic and obstetric factors, as well as the application of a localized food frequency questionnaire to better capture regional dietary patterns. The consistency of findings across linear and logistic models further supports the robustness of the observed associations.

However, several limitations should be acknowledged. First, the cross-sectional design precludes causal inference. Second, self-reported data, including pre-pregnancy weight and dietary intake, may be subject to recall bias. Although the dietary quality score was adapted from an established national dietary assessment framework, the localized version has not undergone formal validation in this population; therefore, some degree of measurement error related to dietary assessment may remain. Future studies using validated dietary instruments and objective dietary assessment methods are warranted to further evaluate its reliability and validity. Furthermore, because self-reported pre-pregnancy weight was used to calculate both pre-pregnancy BMI and PPWR, measurement error in this variable may have contributed to the observed association between pre-pregnancy BMI and PPWR. This finding should therefore be interpreted cautiously and requires confirmation using objectively measured pre-pregnancy weight data.

Third, physical activity was assessed using a questionnaire-based instrument, which may not fully capture activity intensity, patterns, or temporal variations. Fourth, only 21 participants had clinically significant PPWR (≥5 kg). Although a reduced covariate set based on clinical relevance was applied in the sensitivity analysis to minimize the risk of model overfitting, the limited number of outcome events and uneven distribution of some covariates may have affected the stability of logistic regression estimates. Therefore, these findings should be interpreted with caution. In addition, participants were assessed at different postpartum time points (1–3 years postpartum), which may have introduced heterogeneity in PPWR despite adjustment for follow-up duration.

Fifth, because recruitment relied on telephone follow-up, women who could not be contacted or declined participation may have differed from included participants in socioeconomic characteristics, health behaviors, or weight-related outcomes. Therefore, potential selection bias cannot be completely excluded. Sixth, gestational weight gain was analyzed as a continuous variable rather than categorized according to IOM recommendations based on pre-pregnancy BMI categories. Although this approach allowed evaluation of the overall dose-response relationship, it may not fully capture clinically relevant differences in GWG adequacy. Future studies with larger samples should consider incorporating BMI-specific GWG categories to further explore their associations with postpartum weight outcomes.

Finally, although the sample size was adequate for the primary linear regression analyses, the relatively small cohort may have limited statistical power to detect modest associations, particularly for lifestyle-related variables such as physical activity. Future prospective studies with larger sample sizes, standardized follow-up intervals, and objective measurements of dietary intake and physical activity are needed to confirm and extend these findings.

## 5 Conclusion

In this study, higher dietary quality was associated with lower postpartum weight retention among women with a history of GDM living in a sub-high altitude region. This association remained after adjustment for key confounders and was supported by sensitivity analyses. In contrast, physical activity was not independently associated with PPWR after multivariable adjustment. These findings suggest that improving dietary quality may represent a relevant strategy for postpartum weight management in this population. Future longitudinal and interventional studies are needed to further clarify causal relationships and to inform targeted prevention strategies.

## Data Availability

The datasets generated and analyzed during the current study, comprising historical clinical data and telephone follow-up survey data, are not publicly available due to hospital policy and patient confidentiality regulations. However, de-identified datasets supporting the findings of this study may be available from the corresponding author upon reasonable request, subject to approval by the Ethics Committee of Yongping County People’s Hospital.
